# ASIC1a Deficient Mice Show Unaltered Neurodegeneration in the Subacute MPTP Model of Parkinson Disease

**DOI:** 10.1371/journal.pone.0165235

**Published:** 2016-11-07

**Authors:** Daniel Komnig, Silke Imgrund, Arno Reich, Stefan Gründer, Björn H. Falkenburger

**Affiliations:** 1 Department of Neurology, RWTH Aachen University, Aachen, Germany; 2 Institute of Physiology, RWTH Aachen University, Aachen, Germany; 3 JARA BRAIN Institute II, Jülich and Aachen, Germany; Ohio State University, UNITED STATES

## Abstract

Inflammation contributes to the death of dopaminergic neurons in Parkinson disease and can be accompanied by acidification of extracellular pH, which may activate acid-sensing ion channels (ASIC). Accordingly, amiloride, a non-selective inhibitor of ASIC, was protective in an acute 1-methyl-4-phenyl-1,2,3,6-tetrahydropyridine (MPTP) mouse model of Parkinson disease. To complement these findings we determined MPTP toxicity in mice deficient for ASIC1a, the most common ASIC isoform in neurons. MPTP was applied i.p. in doses of 30 mg per kg on five consecutive days. We determined the number of dopaminergic neurons in the *substantia nigra*, assayed by stereological counting 14 days after the last MPTP injection, the number of Nissl positive neurons in the *substantia nigra*, and the concentration of catecholamines in the striatum. There was no difference between ASIC1a-deficient mice and wildtype controls. We are therefore not able to confirm that ASIC1a are involved in MPTP toxicity. The difference might relate to the subacute MPTP model we used, which more closely resembles the pathogenesis of Parkinson disease, or to further targets of amiloride.

## Introduction

Parkinson disease (PD) is a neurodegenerative movement disorder characterized by progressive degeneration of dopaminergic neurons in the *substantia nigra pars compacta* (SNc) and cytoplasmic inclusions of misfolded alpha-synuclein termed Lewy bodies. The cause of cell death in PD is not known and there is currently no causal treatment for PD. Consequently, diverse changes observed in PD are investigated for their potential as a base for neuroprotective treatment strategies.

Inflammation is an important part of the PD pathogenesis. Activation of microglia and increased pro-inflammatory cytokines have been described in PD patients [1–5] and in animal models of PD [6]. Moreover, medication with anti-inflammatory drugs can reduce the risk of developing PD [7]. Inflammation leads to extracellular acidification, and *vice versa* [8]. Accordingly, an increased concentration of lactate has been observed in PD patients using magnetic resonance spectroscopy [9]. This indicates that pH might be more acidic in PD brain.

Administration of the neurotoxin 1-methyl-4-phenyl-1,2,3,6-tetrahydropyridine (MPTP) induces a PD-like pathology in several species, including mice and primates [10, 11]. The consequences of MPTP administration that mimic PD pathogenesis include the inhibition of complex I in the mitochondrial respiratory chain, selective death of dopaminergic neurons, inflammation and an apoptotic cell death mechanism. Interestingly, MPTP administration also leads to lactic acidosis [12].

Acid-sensing ion channels (ASICs) are neuronal, proton-gated cation channels activated by low extracellular pH [13, 14]. ASICs are trimers [15, 16] and in the brain are composed primarily of homomeric ASIC1a, and heteromeric ASIC1a/2a and ASIC1a/2b [17–19]. Consequently, neurons from animals deficient for ASIC1a showed virtually abolished acid-induced currents [20].

Given the evidence for tissue acidification in PD, it is plausible to hypothesize that ASICs might be involved in the pathogenesis of PD. Two studies indeed found that pharmacological inhibition of ASICs reduced neurodegeneration in animal models of PD. In an acute MPTP mouse model, degeneration of dopaminergic neurons was reduced by the nonselective ASIC inhibitor amiloride. Amiloride reduced the degeneration of dopaminergic axon terminals in the striatum and the degeneration of dopaminergic neurons in the SNc in a dose-dependent fashion [21]. The more specific but less tissue permeable ASIC inhibitor psalmotoxin-1 (applied into the cerebral ventricles) slightly reduced the degeneration of dopaminergic axon terminals in the striatum as determined by radiography of the dopamine transporter, but did not rescue the concentration of striatal dopamine, and the degeneration of dopaminergic neurons in the SNc [21]. In addition, amiloride and psalmotoxin-1 (applied intranasally) prevented the progressive degeneration of dopaminergic neurons and axon terminals occurring after intrastriatal injection of 6-hydroxydopamine [22]. ASIC inhibitors have also shown protective effects in models of cerebral ischemia [23, 24], and in models of autoimmune inflammatory disease [25].

To complement these findings with pharmacological inhibitors, we determined MPTP toxicity in mice deficient for ASIC1a, the most important ASIC subunit in neurons of the brain.

## Methods

### Animals and procedures

All mice were housed and handled according to guidelines from the Federation for European Laboratory Animal Science Associations (FELASA). Mice were housed in a pathogen-free facility in a temperature-controlled room (20–24°C) with a 12 h light/dark cycle and with food and water *ad libitum*. The animal experiments were approved by the Animal Care Committee of the RWTH Aachen University and by the District Government of North Rhine Westphalia in Recklinghausen, Germany (No 84–08.05.2013.A056). For all experiments male 10-12-week old ASIC-1a null mutants (ASIC1a^-/-^) and wildtype littermates (ASIC1a^+/+^) were used. The generation of the ASIC1a^-/-^ mice was described previously [20]. ASIC1a^-/-^ mice were backcrossed to C57BL/6 background for more than 8 generations. Mice received either MPTP hydrochloride in 0.9% saline or saline alone. MPTP was administered at a dose of 30 mg free base per kg body weight i.p. at 24 h intervals over 5 consecutive days. MPTP handling and safety measures were in accordance with published guidelines [26]. During the experiment, mice were monitored daily for physical condition and weight loss. All animals that started the experiment survived with <20% weight loss and were included into the analysis. Fourteen days after the last MPTP injection animals were sacrificed by cervical dislocation and brains were prepared for HPLC analysis of catecholamines and immunostaining: After decapitation the left striata were rapidly dissected on ice, quick-frozen in liquid nitrogen, and stored at -80°C until striatal catecholamine concentrations were measured via electrochemical detection. The right striata and the posterior parts of the same brains were fixed for 24 h in 4% PFA, cryoprotected in 30% sucrose for 2 days at 4°C, frozen by immersion in isopentane (-45°C) and then stored at -80°C.

### Immunohistochemistry and quantification

The posterior parts of the brains, including the SN, were serially cut into 30 μm coronal sections. Every third section spanning the SN was stained for tyrosine hydroxylase (TH). The free-floating brain sections were washed three times in Tris-buffered saline (TBS) with 0.1% TritonX (TBS-T). Endogenous peroxidase was blocked to reduce unspecific background by incubation with 0.3% H_2_O_2_ in TBS-T for 30 minutes followed by three washing steps with TBS-T. The primary anti-TH antibody (rabbit polyclonal, Merck Millipore) was incubated overnight at 4°C in a dilution of 1:1,000 in TBS-T containing 3% normal goat serum (Vector Laboratories). The sections were washed again with TBS-T and secondary antibody (biotinylated goat anti-rabbit IgG, Vector Laboratories) was incubated in a dilution of 1:200 in TBS-T for 30 minutes. Subsequently, the sections were washed and incubated with Avidin-Biotin Complex (Thermo Fisher) for 30 minutes, followed by an additional washing step. Visualization of antibody binding was performed via diaminobenzidine (DAB, Vectastain®ABC-Kit Standard PK-4000, Vector Laboratories) in a dilution of 1:20 in PBS for 10 minutes. Sections were mounted on microscope slides. Nissl-counterstaining was performed with haemalaum (VWR) and after dehydration sections were coverslipped with Entellan (Merck Millipore).

TH-positive (dopaminergic) and Nissl-positive (neuronal) cells in the lateral SNc of the right hemisphere were stereologically counted using the optical fractionator method (StereoInvestigator v11, MicroBrightField) as described previously [27]. In brief, neurons were manually indentified in 50 x 50 μm counting frames presented by the software using an Axioskop 2 microscope (Carl Zeiss Vision) and an oil immersion 63x objective (NA 1.4). Grid size was 50 x 50 μm, and every third section was analyzed. Counts were performed blinded for genotype and treatment.

### Catecholamine analysis

Catecholamines were measured by HPLC with electrochemical detection as described previously [27–29]. Striatal tissue was homogenized in 50 μl of 0.1 M perchloric acid per mg of striatal tissue. Cell debris was pelleted by centrifugation (17,000 x g for 20 min at 4°C). 20 μl of supernatant was injected onto a C18 reverse-phase column (Prontosil 120-3-C18, Thermo Fisher). The mobile phase consisted of 85 mM sodium acetate, 35 mM citric acid, 0.5 mM octane sulfonic acid, 0.15 mM EDTA solution and 10% methanol (pH 4.3). Flow rate was 0.8 ml/min. Electrochemical detection of dopamine, DOPAC (3,4-dihydroxyphenylacetic acid) and homovanillic acid concentrations was carried out at 800 mV. Calibration was achieved by comparison with external standards run after every third sample and Chromeleon 6.80 software (Thermo Fisher Scientific). Values are represented as ng catecholamine per mg wet weight tissue.

### Statistical analyses

Data are presented as mean ± SEM with "n" equal to the number of animals. Statistical analyses were performed using GraphPad Prism 5.0 (GraphPad Software). The statistical test for comparison of treatment and genotype was 2-way ANOVA followed by Bonferroni posthoc test or by unpaired t-test as indicated in the text. The null hypothesis was rejected at the 0.05 level.

## Results and Discussion

**Fig 1:**
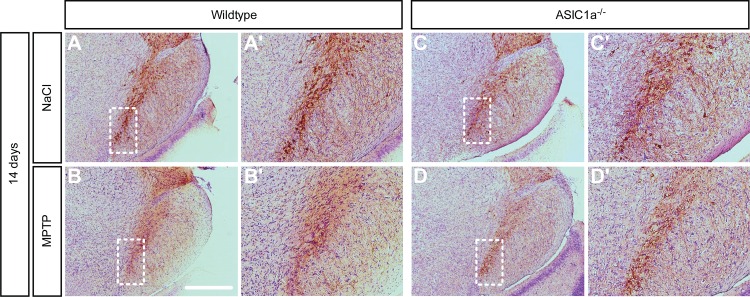
MPTP-induced loss of TH positive neurons in the *substantia nigra pars compacta*. (A-D) Representative images of dopaminergic neurons stained for tyrosine hydroxylase (TH) in coronal midbrain sections of ASIC1a^+/+^ and ASIC1a^-/-^ mice 14 days after MPTP or saline treatment. The framed area of the lateral SNc represents the area analyzed by the stereological countings depicted in Fig 2. Scale bar 100 μm. (A´-D´) Higher resolution images of A-F.

**Fig 2:**
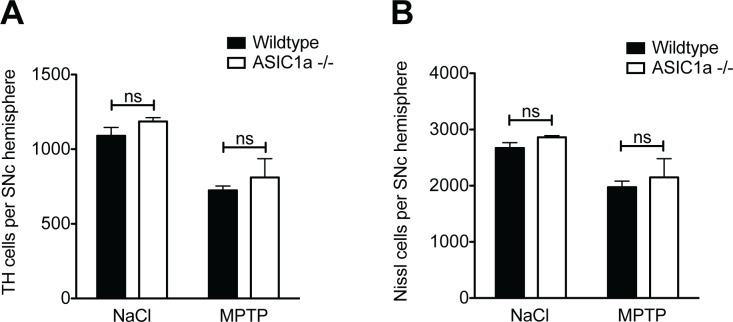
Loss of ASIC1a did not affect neurodegeneration after MPTP. Mice received either subacute MPTP treatment or saline only. Dopaminergic (TH-positive) cells (A) and Nissl-positive cells (B) were stereologically counted in one hemisphere of the SNc after 14 days. Results were analyzed by two-way ANOVA followed by Bonferroni post hoc tests; ns = non significant. Number of animals: NaCl ASIC1a^+/+^: n = 3; NaCl ASIC1a^-/-^: n = 3; MPTP ASIC1a^+/+^: n = 5; MPTP ASIC1a^-/-^: n = 4.

**Fig 3:**
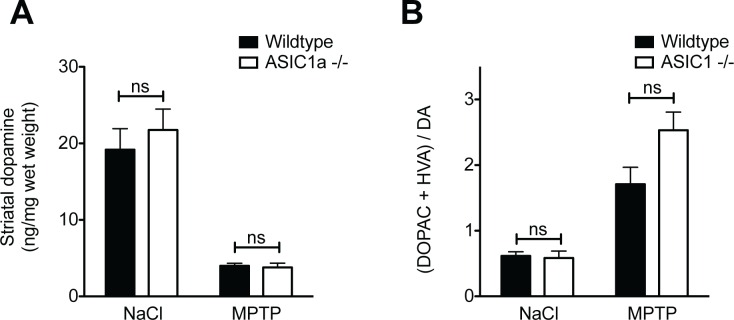
Loss of ASIC1a had no impact on striatal catecholamines after subacute MPTP treatment. Striatal concentration of dopamine (A) and the metabolite ratio (B) were determined by HPLC. Results were analyzed by two-way ANOVA followed by Bonferroni post hoc tests; ns = non significant. Number of animals: NaCl ASIC1a^+/+^: n = 3; NaCl ASIC1a^-/-^: n = 3; MPTP ASIC1a^+/+^: n = 5; MPTP ASIC1a^-/-^: n = 4.

We first compared the dopaminergic system of mature, age-matched ASIC1a^-/-^ and ASIC1a^+/+^ mice not receiving MPTP. There was no difference in the number of dopaminergic neurons in the SNc, as identified by staining for tyrosine hydroxylase (TH), between the two genotypes (ASIC1a^+/+^ 1090 ± 56, ASIC1a^-/-^ 1186 ± 25; p = 0.19, unpaired t-test; Figs 1A, 1C and 2A). Furthermore, there was no difference in the concentration of striatal dopamine between ASIC1a^+/+^ and ASIC1a^-/-^ mice (ASIC1a^+/+^ 19.17 ± 2.8 ng/mg wet weight, ASIC1a^-/-^ 21.75 ± 2.75 ng/mg wet weight; p = 0.54, unpaired t-test; Fig 3A). Taken together, genetic depletion of ASIC1a had no impact on the number of dopaminergic neurons in the SNc and on dopamine levels in the striatum of C57BL/6 mice.

To model PD we chose the subacute MPTP paradigm with 5 injections of 30 mg per kg, applied i.p. 24 h apart. Animals were analyzed 14 days after the last MPTP injection. There was a robust decline in the number of dopaminergic neurons in ASIC1a^-/-^ and ASIC1a^+/+^ animals (Figs 1B, 1D and 2A). The decline was similar in both genotypes with (34% and 32%; Fig 2A; 2-way ANOVA: significant effect of MPTP, n.s. interaction, n.s. difference between genotypes). To demonstrate that the decline in TH positive neurons really results from cell death and not merely from downregulation of TH, we also quantified the number of Nissl-positive neurons in the SNc. The results were similar as for TH-positive neurons (reduction by 26% and 25%; Fig 2B).

Similar to the number of TH-positive neurons, striatal dopamine was strongly reduced in ASIC1a^-/-^ and ASIC1a^+/+^ animals 14 days after MPTP administration (79% versus 83%, Fig 3A). Again there was no difference between the two genotypes (2-way ANOVA: significant effect of MPTP, n.s. interaction, n.s. difference between genotypes). Dopamine deficiency was partially compensated by an increased amount of the dopamine metabolites homovanillic acid (HVA) and 2,4-dihydroxyphenylacetic acid (DOPAC) relative to dopamine (Fig 3B). There was a trend for more metabolites in ASIC1a^-/-^ mice, which could indicate a requirement for more dopamine turnover, but this effect was not statistically significant (Fig 3B).

The similarity between ASIC1a^-/-^ and ASIC1a^+/+^ animals in the MPTP model was unexpected given the protective effect of amiloride in previous studies [21, 22]. In our experiments, both genotypes showed a reduction of dopaminergic neurons and striatal dopamine, which was similar in extent to the reduction in previous work with the same paradigm [27, 29], indicating that the MPTP model was induced successfully. Currents elicited by acidic pH are nearly abolished in the ASIC1a deficient mice we used [20], making it unlikely that other ASIC subunits compensated for the deficiency of ASIC1a. However, compensatory changes in ASIC1a deficient mice cannot be ruled out entirely.

Amiloride not only blocks ASICs, but also a number of other membrane proteins that might be involved in neurodegeneration in the MPTP mouse model, including the Na^+^/Ca^2+^ exchanger (NCX) [30]. NCX could be involved in the degeneration of dopaminergic neurons following MPTP administration given the important role of calcium homeostasis in this model [31–33] and the involvement of NCX in a variety of disease states [34]. Indeed, SEA0400, a specific NCX inhibitor was recently shown to reduce dopaminergic neurotoxicity in the MPTP model [35]. Yet, the inhibition of NCX by amiloride cannot explain the protection of dopaminergic neurons in the 6-hydroxydopamine model by psalmotoxin-1, a specific inhibitor of ASIC1a homomers [22].

Furthermore, the discrepancy may result from differences between the PD models used. Arias and colleagues used a single dose of MPTP (40 mg/kg) administered two hours after amiloride, whereas a subacute MPTP regimen (5x30 mg/kg) was used in this study. Oxidative stress, excitotoxicity and non-apoptotic cell death are more pronounced in the single dose MPTP paradigm [36, 37], whereas apoptotic cell death predominates in the subacute paradigm [38, 39]. It is possible that ASICs are more relevant in the acute MPTP paradigm than in the subacute MPTP model we used. Since PD is a chronic disease with apoptotic cell death, the subacute MPTP paradigm more closely resembles disease pathogenesis. Consequently, our findings could not confirm that ASICs are a promising therapeutic target for PD.

## Abbreviations

ASIC: acid-sensing ion channel
DOPAC: 3,4-dihydroxyphenylacetic acid
HVA: homovanillic acid
MPP^+^: 1-methyl-4-phenylpyridinium
MPTP: 1-methyl-4-phenyl-1,2,3,6-tetrahydropyridine
PD: Parkinson disease
SN: *substantia nigra*
SNc: *substantia nigra pars compacta*
TH: tyrosine hydroxylase
